# Advances in proteomics: characterization of the innate immune system after birth and during inflammation

**DOI:** 10.3389/fimmu.2023.1254948

**Published:** 2023-10-06

**Authors:** Tue Bjerg Bennike

**Affiliations:** Medical Microbiology and Immunology, Department of Health Science and Technology, Aalborg University, Aalborg, Denmark

**Keywords:** plasma, mass spectrometry, complement system, neutrophils, neutrophil extracellular traps, ontogeny, rheumatoid arthritis, inflammatory bowel disease

## Abstract

Proteomics is the characterization of the protein composition, the proteome, of a biological sample. It involves the large-scale identification and quantification of proteins, peptides, and post-translational modifications. This review focuses on recent developments in mass spectrometry-based proteomics and provides an overview of available methods for sample preparation to study the innate immune system. Recent advancements in the proteomics workflows, including sample preparation, have significantly improved the sensitivity and proteome coverage of biological samples including the technically difficult blood plasma. Proteomics is often applied in immunology and has been used to characterize the levels of innate immune system components after perturbations such as birth or during chronic inflammatory diseases like rheumatoid arthritis (RA) and inflammatory bowel disease (IBD). In cancers, the tumor microenvironment may generate chronic inflammation and release cytokines to the circulation. In these situations, the innate immune system undergoes profound and long-lasting changes, the large-scale characterization of which may increase our biological understanding and help identify components with translational potential for guiding diagnosis and treatment decisions. With the ongoing technical development, proteomics will likely continue to provide increasing insights into complex biological processes and their implications for health and disease. Integrating proteomics with other omics data and utilizing multi-omics approaches have been demonstrated to give additional valuable insights into biological systems.

## 1 Introduction

Proteins are products of our transcribed and translated genome and are the effector molecules for the majority of cellular functions. Proteomics is the large-scale identification and relative quantification of proteins, peptides, and post-translational modifications (PTMs), and can cover the study of all expressed proteins by an organism, i.e. the proteome. Liquid chromatography mass spectrometry (LC-MS/MS) remains the most widely used core technology in proteomics for large-scale characterization of the proteome. The versatile method can analyze a wide variety of sample types and provide comprehensive characterization of the proteome. In the context of this review, "proteomics" refers to identification and relative quantification of peptides and proteins by LC-MS/MS.

The proteomics workflows have undergone continuous advancements, especially in terms of sample preparation and speed and sensitivity of the platforms largely driven by the technical development and novel MS data acquisition and sample preparation methods. In the early 2010s, the typical sample preparation prior to analysis took one to two days. Proteins in the thousands could be measured in tissue samples in the course of 3-4 hours of LC-MS/MS analysis time (1–3). Today, parallel sample preparation in 96-well plates enable rapid processing of hundreds of samples in a matter of hours rather than days supporting large scale studies (4, 5). The analysis of more than 1,000 plasma samples have made it possible to study the effect of weight loss and maintenance, and associate it with a reduction of biological pathways related to inflammation and activation of lipid metabolism (6, 7). In tissue samples, over 10,000 proteins can be identified in less than two hours (8, 9). As an example, the methodology has been applied to identified altered protein levels during testis cancer and link downregulation of the complement system, likely to evade the innate immune response, with the pathogenesis of the disease (8). Identification of cancer-specific biomarkers, e.g. proteins which leak from the tumor microenvironment to the blood, holds great potential for noninvasive diagnostics.

Advancements in proteomics have found broad applications, amongst others in the field of immunology to study the innate immune system humoral and cellular components in health and disease. Traditionally, 'humoral' described antibody-mediated adaptive immunity. However, the term has now been expanded to include the soluble components of both the innate and adaptive immune system (10). This review highlights recent developments in proteomics sample preparation protocols and MS methods and provides examples of recent discoveries within the innate immune system in health and disease driven by proteomics. The aim is to provide the reader with an overview of currently available methods and proteomics capabilities to study the innate immune system.

Section 2 provides a brief overview of humoral components of the innate immune system in the blood and neutrophile granulocytes, including neutrophil extracellular traps (NETs) and the formation by NETosis. The levels of humoral components change during early development, infections, and during inflammatory diseases, and may provide a perspective on ongoing innate immune system processes and identify biological targets with translational potential for guiding diagnosis and treatment decisions.

Section 3 describes the main technical challenges in plasma proteomics, and the principle of the LC-MS/MS proteomics workflow. The section emphasizes advancements in workflows aimed at increasing the coverage of the plasma proteome to amongst others study the humoral innate immune system components.

Section 4 highlights recent applications of plasma proteomics to study the developmental trajectory in levels of the humoral innate immune system components during the critical first week after birth, which plays a vital role in newborn immunity (11).

Finally, section 5 discusses the role of NETs in inflammatory diseases and its potential as a therapeutic target. NETs are generated by the most abundant innate immune cell, the neutrophils, and are increasingly recognized as a major contributor to the onset and persistence of prevalent inflammatory diseases (12, 13), including rheumatoid arthritis (RA) and inflammatory bowel disease (IBD) (3), and have been associated with cancers (14).

## 2 A brief overview of the innate immune system

The innate immune system comprises cellular components (including neutrophils and monocytes/macrophages) and humoral components (including the complement system and acute phase proteins). It is the first line of defense against infections. Breach of the physical/chemical barrier or invasion of pathogens will initiate an innate immune response within minutes to hours. The rapid response is facilitated by the persistent availability of the innate immune components in blood, which are continuously synthesized irrespective of any pathogen encounter (15). Consequently, the pattern recognition receptors (PRRs) in the innate immune system have an affinity to conserved pathogen-associated molecular patterns (PAMPs), and re-exposure to a given pathogen results in the same innate immune response. This is in contrast to the adaptive immune response which is modulated upon re-exposure (16, 17). Most of the humoral innate immune system components are protein-based and can be studied by proteomics to characterize the innate immune response in diseases.

### 2.1 The complement system

The complement system is part of the first innate immune response against invading pathogens and include more than 30 proteins circulating in the plasma, in addition to complement receptors on immune cells including neutrophils and monocytes/macrophages. Similar to the coagulation system in blood, the complement system utilizes serine proteases which are sequentially activated in amplification cascade reactions thereby allowing binding of few PAMPs by PRRs to initiate a vast response (18).

**Figure 1:**
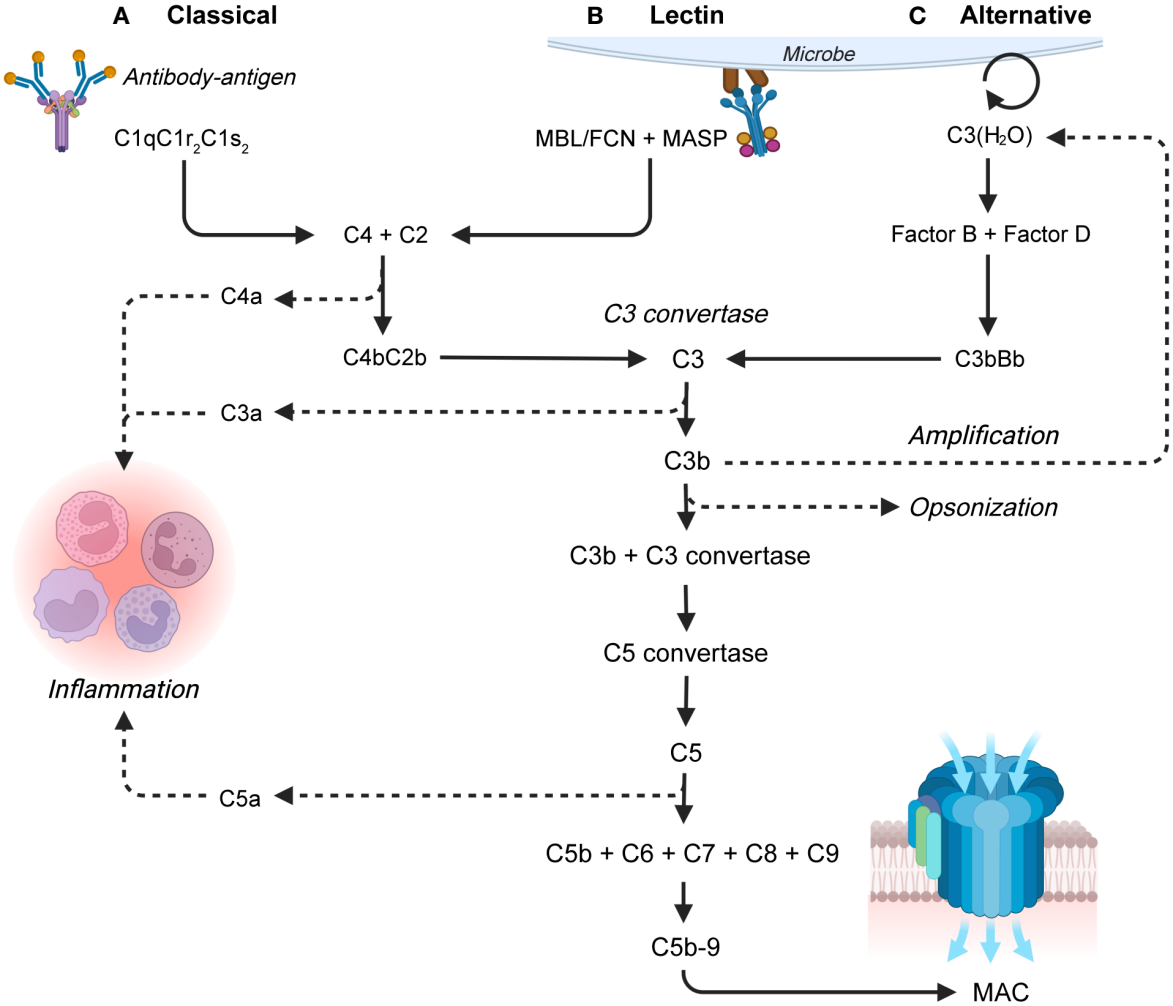
Overview of the complement system, with a focus on the three activation pathways: **(A)** The classical pathway which is activated by antibody-antigen complexes, **(B)** the lectin pathway which is activated on bacterial and viral surfaces, and **(C)** the alternative pathway which is continuously active. The pathways lead to the terminal pathway and formation of the membrane attack complex (MAC). The larger proteolytic fragment of C2 is in this review termed C2b by the recently recommended nomenclature for complement pathways which aligns C2 with the other complement factors (19). FCN, ficolin; MBL, mannan-binding lectin; MASP, mannan-binding lectin serine proteases (20).

The complement system can be activated by three major pathways. The classical pathway (**Figure 1A**) can be initiated by binding of complement component (C)1q to antigen-antibody immune complexes, consisting of multiple proximate immunoglobulin (Ig)G or few IgM molecules (21, 22). The lectin pathway (**Figure 1B**) can be initiated by binding of ficolins (FCN), mannose-binding lectin (MBL), and collectins to carbohydrates or N-acetyl-glucosamine exposing groups found on the surface of many bacteria and viruses (23, 24). Activation of the classical and lectin pathways lead to cleavage of C4 by the C1-complex (C1qC1r_2_C1s_2_) or mannan-binding lectin serine proteases (MASP) 1 and 2, respectively. C4 is cleaved into the anaphylatoxin C4a and a larger C4b fragment. C4b binding of C2 enables cleavage into a C2a fragment and a larger proteolytic C2b, which remains bound to C4b and form a C3-convertase (C4bC2b) (25, 26). The alternative pathway (**Figure 1C**) is continuously active in low levels due to hydrolysis of the C3-thioester group resulting in formation of C3(H_2_O) with a "C3b-like" activity. Binding of factor B (27) and cleavage of the complex by factor D form a C3-convertase (C3bBb). The C3bBb-complex can be stabilized by binding to properdin (also known as complement factor P, CFP), the only known complement activation enhancer (28).

All three activation pathways result in cleavage of C3 into the anaphylatoxin C3a and the opsonin C3b. In addition to spontaneous activation, the alternative pathway can amplify complement activation by producing additional alternative C3 convertases using C3b generated by either pathway (28). High local concentrations of C3b initiates the terminal pathway, where C3b associates with a C3 convertase and forms a C5 convertase complex. The C5 convertase initiates the formation and assembly of the membrane attack complex (MAC), by cleaving C5 into the anaphylatoxin C5a and C5b. C5b recruits C6, C7, and C8, and induces C9 polymerization, thereby generating a C5b-C9 transmembrane MAC pore structure (29). The MAC causes bacterial lysis capable of eliminating certain pathogens (30). In addition to MAC formation and opsonization, complement induces several pro-inflammatory effects many of which are induced by the complement anaphylatoxins (C3a, C4a, and C5a). The effects include chemotaxis, synthesis of reactive oxygen species (ROS) in neutrophils and monocytes/macrophages, and histamine release from mast cells and vasodilation (29, 31).

### 2.2 Neutrophils and neutrophil extracellular traps

The immunological actions by the complement system works in synergy with a variety of immune cells, including neutrophils and monocytes/macrophages. Neutrophils represent the most abundant circulating immune cells and the first cells recruited from blood. The cells are recruited by cytokines and anaphylatoxins to the site of inflammation where the immunological actions include secretion of inflammatory proteins, ROS, and phagocytosis (32). Stimulation by C5a causes neutrophils to release C3, complement factor B (CFB), CFP, and enhance local complement system activation (33). Neutrophils may also release NETs in response to large pathogens (34) or specific stimuli including gram-positive and gram–negative bacteria, fungi, lipopolysaccharide (LPS), and IL-8 (35).

NETs were first described in 2004 and are web-like structures with a backbone of decondensed chromatin fibers coated with antimicrobial proteins (36). NETs are important for immunity and are released in a cell process termed NETosis. Initiation results in activation of NADPH-oxidase (NOX2). NOX2 generates intracellular ROS which increases the nucleus membrane permeability, leading to activation and translocation of granular neutrophil elastase to the nucleus. In the nucleus, neutrophil elastase partially degrade specific histones resulting in chromatin decondensation, enhanced by myeloperoxidase (MPO). Increased intracellular calcium levels during NETosis activate the enzyme peptidyl deiminase (PAD) 4. PAD4 localizes to the cell nucleus and catalyzes the deimination of arginine amino acids into citrullines on histone H3 proteins (37). Citrullination reduces the positive charge of proteins (38) and relaxes the chromatin binding by histone H3, thereby causing further chromatin decondensation. The neutrophil nuclear envelope disassembles and nuclear decondensated chromatin mixes with cytoplasmic and granule components (39). Finally, the plasma membrane permeabilizes, and NETs coated with proteases and antimicrobial peptides and proteins expand into the extracellular space (40, 41). Different processes of NETosis exists, where not all results in the immediate death of the neutrophil (42). The NETs trap and neutralize pathogens, and neutrophil elastase has been found responsible for the majority of the proteolytic activity (43). The anti-microbial effect is enhanced by NET-bound CFP, produced by neutrophils, which enables NETs to augment complement activation and induce MAC formation via the alternative pathway (33). In this way, neutrophils and NETs work in synergy with the complement system to eliminate pathogens.

### 2.3 Acute phase proteins

Several of the pro-inflammatory cytokines and chemokines that are released during infection and inflammation stimulate hepatic synthesis and circulatory secretion of acute phase proteins (APP). APP are defined as proteins where the serum concentration change by >25% during inflammation (44). Interleukin (IL)-6 is widely viewed as the major inducer of APP synthesis, but APP-synthesis can also be stimulated by e.g. IL-1, tumor necrosis factor alpha (TNF-α), interferon gamma (IFN-γ), and transforming growth factor beta (TGF-β) (45). The positively regulated APP increase during infection and inflammation and most are involved in the innate immune response. These include complement components (C3, C4, C9, and factor B), serum amyloid A (SAA1), C-reactive protein (CRP), and transport proteins including haptoglobin (HP) (44).

SAA1 and CRP are among the APP with the highest increase during infection (46). SAA1 has a wide range of inflammatory functions for priming neutrophils and increasing the production of ROS. CRP binds peptides on bacterial surfaces and can activate complement by C1q binding thereby contributing to inflammation (47, 48). Additionally, CRP on bacterial surfaces can bind to receptors for the Fc portion of IgG and perform opsonization of pathogens (49), and stimulate generation of additional pro-inflammatory cytokines that enhance the inflammatory response (47, 48). CRP blood levels are today widely used as a non-specific but clinically useful marker for infection (50).

HP is a scavenger protein which binds free hemoglobin in plasma which is an oxidizing agent causing tissue damage. In addition, bacteria can obtain iron from the heme group to increase proliferation. HP binding of hemoglobin reduce the available iron for bacterial growth and HP can bind CD11b/CD18 integrins on neutrophils and regulate the activity of the immune system, thereby asserting an anti-bacterial effect. Thus, HP has both antioxidant, antimicrobial, and anti-inflammatory properties (32, 51), and increased plasma levels have been reported during infection and inflammation (52) and in newborns in the days after birth (53).

## 3 Plasma proteomics to study the humoral innate immune system

**Figure 2:**
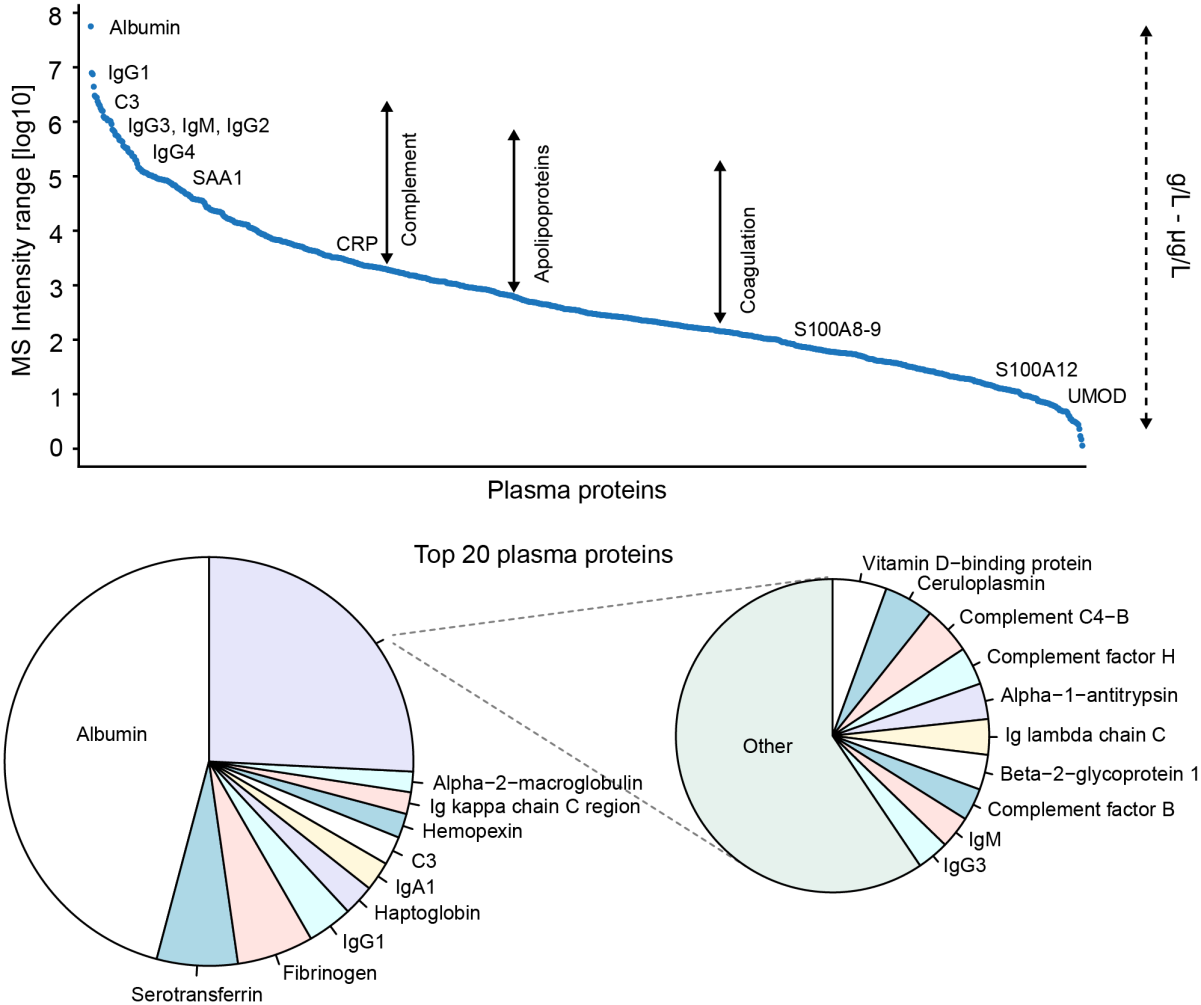
Proteome composition of blood-plasma ranked based on MS signal intensities. **(A)** Top 1000 and **(B)** top 20 plasma proteins identified in adult plasma by proteomics. Proteomics data from Bennike et al. (4). A mapping of known absolute protein concentrations against averaged data from The Plasma Proteome Database demonstrates that the identified proteins include proteins with plasma concentrations from μg/L to g/L. NB the plasma protein MS signal intensity ratio and rank may be different compared to the absolute concentration.

Blood-plasma is an easily obtainable material with a high protein concentration of 50-70 g/L (54). Over 5,000 proteins in plasma have been identified by proteomics (55, 56) demonstrating an impressive complexity of the plasma proteome. The proteins include the majority of the innate immune system components and signaling molecules that circulate in the blood (**Figure 2A**). Monitoring the levels of these components may be used to characterize ongoing innate immune reactions and inflammatory processes in inflammatory diseases. As an example, Bennike et al. have applied plasma proteomics to study the relative levels of the complement system components in the first week after birth (53). In addition, inflammatory processes are linked with cancer (57), and proteomics identification of cancer biomarkers with diagnostic and prognostic value in plasma holds vast potential to guide diagnosis and therapy decisions (57).

Proteomics thereby seems promising as a methodology to identify novel blood-based markers with translational potential for diagnostics and prognostics. However, relatively few biomarkers identified by proteomics have progressed to be used in clinical practice (58). This is likely a consequence of the protein composition of plasma, and plasma-derived body fluids such as synovial fluid, which presents several technical challenges for plasma proteomics, resulting in a lack of sensitive, reproducible, and high-throughput compatible proteomics workflows. The concentration range of the plasma proteins cover a vast dynamic range of 11-12 orders of magnitude (54), by far exceeding the dynamic range of modern mass spectrometers (59). In addition, the distribution of the protein concentrations is highly skewed, with the 22 most abundant proteins, including albumin, Ig's, and C3, constituting 99% of the total protein amount (54). The relatively few highly abundant proteins hinder the proteomics-based detection and quantification of the lower abundant proteins (**Figure 2B**), effectively reducing the number of detectable and quantifiable plasma proteins (4).

**Figure 3:**
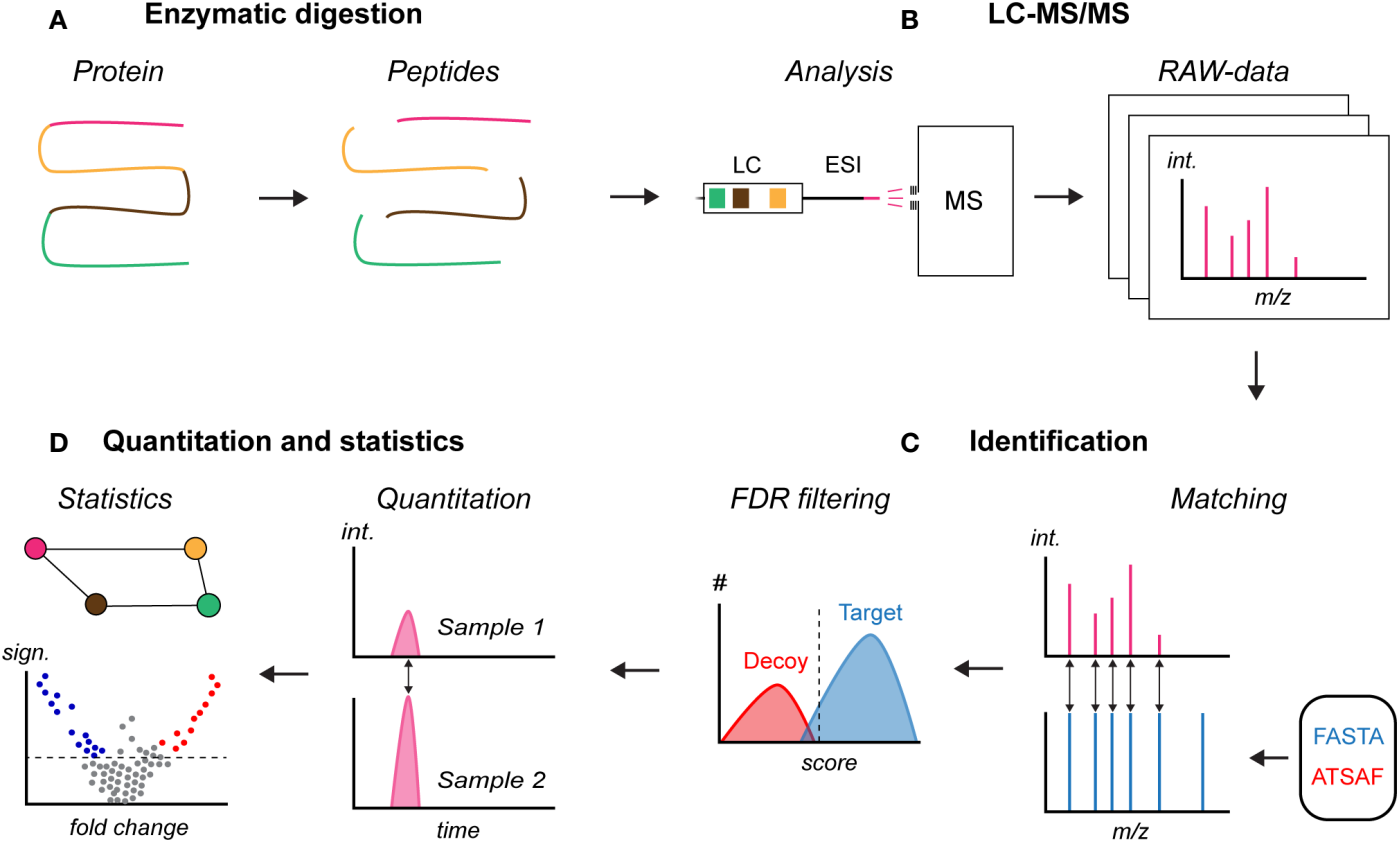
Principle of bottom-up proteomics. **(A)** Enzymatic digestion of a denatured and solubilized protein sample, **(B)** liquid chromatography mass spectrometry (LC-MS/MS) analysis of the peptide sample, **(C)** identification of peptides and inferring of proteins in the sample based on a user-provided FASTA database which also can include a spectral library with measured peptide fragmentation signals, and **(D)** relative quantification and identification of proteins with a statistically significant change. FASTA, protein database; ATSAF, reversed protein database for decoy; #, number of matches; int., signal intensity; sign., statistical significance.

This section discusses the widely used bottom-up proteomics workflow and addresses recent developments with a focus on improving the coverage of the plasma proteome (**Figure 3**, will be used throughout this section). Bottom-up proteomics refers to the analysis of samples where the proteins have been enzymatically proteolyzed into peptides prior to LC-MS/MS analysis (**Figure 3A**) (60). While mass spectrometers can measure the mass of intact proteins, analysis on the peptide level has several advantages over "top-down" proteomics, including an increased efficiency in obtaining amino acid sequence information. This is especially true for complex protein mixtures, e.g. from biological samples (61).

### 3.1 Sample preparation for bottom-up proteomics

A vital part of experimental designs is randomization of the sample order. Sample handling and processing can introduce differences between the samples. If the samples are batched (grouped) and processed condition-wise without randomization, the unintentionally introduced technical variance can be inseparable from the biological variance of interest. Such batch effects can bias the interpretation of the results which can lead to false conclusions. In proteomics, systematic differences leading to batch effects can be introduced at several steps, including during sample handling, sample preparation, and LC-MS/MS analysis where the performance often changes over time due to amongst others column wear, drift in calibration, temperature fluctuations, and contaminations. A complete randomization of the sample order can, however, create imbalanced designs by mere chance, where all samples of a given condition are batched. This can be overcome by block randomization, where the samples are batched into blocks according to a known grouping. While recommended, block randomization has yet to find widespread usage by the proteomics society (62).

The first step in preparing a sample for bottom-up proteomics in a generic workflow is solubilization of the proteins and inhibition of *in vivo* protease activity. Plasma and plasma-derived body fluids can often readily be mixed with a lysis buffer containing detergents to solubilize the proteins. Homogenization of solid samples, such as tissues or cells, is critical to ensure optimal proteome coverage, and can be done mechanically in a lysis buffer. To prevent unintended proteins degradation by *in vivo* proteases, and ensure a later efficient enzymatic digestion, the proteins are denatured by reducing the cysteine disulfide bonds with dithiothreitol (DTT) or tris(2-carboxyethyl)phosphine (TCEP), which often is included in the lysis buffer. The denaturation can be enhanced by heating. To prevent reformation of the disulfide bonds which would complicate the downstream data-analysis, reduction is followed by alkylation, commonly with iodoacetamide (IAA) (63). The denatured and solubilized protein sample is subsequently proteolyzed into peptides. Commonly the protease trypsin is used which cleaves the peptide bond on the C-terminal side after arginine and lysine. The enzymatic activity of trypsin is usually high, but can be reduced, e.g. in the presence of a proline on the C-terminal side of arginine or lysine residues (64) or upon modifications at or close to the cleavage site, such as, citrullination of arginine as shown by Bennike et al. (38). Following digestion, the peptide-sample can be purified from salts and contaminants using C18 columns, either offline or automatically in combination with the LC-MS/MS analysis (63). No universally applicable method for protein digestion has been developed, and the recent development in available methods are discussed in the following sections.

#### 3.1.1 In-solution digestion

In-solution is arguably the simplest digestion method, where all chemicals and components are sequentially added to a tube. The usage of a single tube minimize protein loss caused by adsorption to the tube and tip walls. However, given the absence of buffer exchange or wash steps all chemicals must be compatible with the digestion conditions and downstream analysis, and C18 purification is often required to remove contaminants from the peptide product. In addition, the enzymatic digestion efficiency is often reduced leading to a decreased number of identified peptides and proteins as shown by Bennike et al. and others (65, 66).

#### 3.1.2 In-gel digestion

The in-gel digestion protocol was introduced by Shevchenko et al. in 1996 (67) and still has widespread usage due to its many advantages. Briefly explained, the homogenized and solubilized protein sample is pre-separated by one- or two-dimensional gel electrophoresis on polyacrylamide gels, which efficiently remove low molecular weight impurities. The purification enables the use of detergents and buffer components which are incompatible with downstream analysis. The gels are stained, often by silver-staining or Coomassie brilliant blue, bands of interest are excised from the gel, enzymatically digested, and analyzed by LC-MS/MS (67, 68). In-gel digestion used to be a cornerstone for proteomics research as a well-established and robust method. However, in spite of the many advantages of in-gel digestion, the method has several drawbacks. These include a relatively laborious and time-consuming protocol (5) with a high risk of keratin contamination from the researcher and a lower reproducibility in protein identifications (65, 69), compared to other methods.

#### 3.1.3 Gel-free digestion methods for proteomics

A paradigm shift was the introduction of sample preparation and digestion for proteomic analyses using spin filters termed filter-aided sample preparation (FASP), which overcomes many of the limitations from the in-gel and in-solution protocols. The FASP method was described by Manza *el at* in 2005 (69), fully realized by Wiśniewski et al. in 2009 (70), and further improved by Leon et al. in 2013 (66).

Briefly explained, solubilized proteins are transferred to a molecular cutoff ultrafiltration unit. All reactions are done on the filter which facilitates buffer exchanges and washes. The volume during digestion can be kept at a minimum which increases the protease concentration and digestion efficiency. Compared to the in-gel digestion, the hands-on and total sample preparation time is reduced, and the method has a lower risk of keratin contamination from the researcher. As shown by Bennike et al. and others, FASP has a higher reproducibility in protein identifications, compared to in-gel and higher number of identified proteins as compared to in-solution (65, 69). The FASP protocol has been extensively used to characterize the innate immune system in health and diseases for a variety of sample-types, including plasma, plasma-derived synovial fluid (38, 65), and tissue (3).

A drawback of FASP is the relatively large loss of sample material to the FASP filters. While plasma studies using blood drawn with syringes are not sample limited, studies of finger- or heel-prick samples, e.g. of newborn blood or isolated immune cells, may benefit from protocols requiring less material. To improve the proteome coverage of sample-limited studies, the in-StageTip (iST) method by Kulak et al. (71) and suspension trap (S-trap) method by Zougman et al. (72) were developed and published in 2014. Rather than using the larger spin-filers in FASP, iST employs a C18 disk inserted into a pipette tip container and S-trap uses several disks of different filters to retain proteins and peptides and facilitate C18 purification. Another widely used method by Hughes et al. (73, 74) termed single-pot solid-phase-enhanced sample preparation (SP3) relies on binding the proteins and peptides on the hydrophilic surface of carboxylate-coated magnetic beads. Washes and buffer exchanges are performed while retaining the beads using a magnet. The usage of small disks or beads compared to the relatively large FASP spin-filters minimizes sample loss. A direct comparison of iST, SP3, and FASP showed similar performances for 20 μg of starting material, although handling time was reduced with iST and SP3 (75). However, below 10 μg of starting material, the number of identified proteins and the quantitative reproducibility decrease for FASP. In contrast, iST and SP3 provide high proteome coverage down to 1 μg of starting material (75). Geyer et al. (76) performed a proof-of-concept study using iST with 5 μL blood collected from a single finger prick and identified 285 proteins across ten individuals, which could be beneficial where normal blood-draw by syringe may be challenging, e.g. in studies of newborns. However, the protocol has yet to find wide usage.

#### 3.1.4 Parallel and automated digestion methods

An increasing number of proteomics studies include hundreds to thousands of samples (7, 77, 78). In translational and clinical proteomics with large cohorts, the multi-tier plate is the preferred format for sample processing and storage. The plates facilitate the usage of multi-channel pipettes and are more easily automated (5).

A 96-well plate format of the FASP protocol was published in 2013 (79). However, the several centrifugation steps needed for liquid transfer during buffer changes could only be performed at 2,200×g which, compared to the original FASP protocol with 14,000×g, prolonging all centrifugation steps from tens of minutes to three to four hours. To decrease the sample preparation time while keeping the required amount of sample low, the MStern digest method was developed and published by Berger et al. in 2015 (5), and modified for plasma by Bennike et al. in 2018 (4, 80). The MStern protocol employs large pore polyvinylidene difluoride (PVDF) membranes in a 96-well plate for protein retention, thereby utilizing the protein charge interactions known from western blotting and vacuum for the liquid transfer steps (5). The MStern protocol is a simple and fast method and has e.g. been applied by Boada et al. (81) to study host response pathways during virus infections, by Bennike et al. (4) to study the impact of surgery on the serum proteome, and by Bennike et al. (53, 82) to study the development of the complement system over the first week after birth. Similarly, a 96-well plate format of the SP3 protocol has been developed and was in 2020 automated (83). The MStern protocol results in a higher ratio of tryptic missed cleaves which may increase the repeatability in protein identifications across samples as additional peptides can be generated from each protein (5). However, the missed cleavages may be problematic e.g. when studying the PTM citrullination. The high efficiency of trypsin to cleave after arginine amino acids, is inhibited by arginine citrullination, and as demonstrated by Bennike et al. (38), a tryptic missed cleavage after arginine can be used to support identification of citrullinated peptides. However, due to the higher number of peptides with missed cleavages with the MStern protocol, a higher ratio of peptides falsely annotated as citrullinated can be expected with this protocol. Although this has yet to be experimentally demonstrated, studies of citrullination will likely benefit from the iST or SP3 protocols. A comparison between the iST, SP3 and MStern digest protocols found the highest number of identified proteins with iST and SP3 (84). However, the study used urine as material for the evaluation, and a comparison between the 96-well plate digest protocols remains to be performed using plasma which would be beneficial for large-scale plasma proteomics studies.

### 3.2 Proteomics analysis using LC-MS/MS

Following protein digestion, the generated peptide sample is analyzed by LC-MS/MS (**Figure 3B**). In short, the peptides are introduced onto a reversed-phase LC column. The column contains a stationary phase of hydrophobic C18 material, which binds the peptides. A mobile phase is passed through the column consisting of water mixed with acetonitrile acidified with formic acid. By gradually increasing the acetonitrile-to-water ratio from approximately 2% to 40% over minutes to hours, the hydrophobicity of the mobile phase can steadily be increased. At a given hydrophobicity, which is dependent on the peptide sequences and modifications, the peptides will pass from the stationary phase into the mobile phase and be eluted from the column. The eluting peptides are ionized and introduced into the mass spectrometer using electrospray ionization (ESI) (85), and the mass spectrometer measures the mass-to-charge ratio (m/z) of the peptides and/or peptide fragments and the signal intensities (61, 86). Lighter ions or ions with a higher charge will have a lower m/z value, while heavier ions or ions with a lower charge will have a higher m/z value. The signal intensity is dependent on the concentration, but also the properties of the peptides, including amino acid sequence and modifications. Resultingly, two peptides with distinct amino acid sequence can yield different signal intensities despite having the same concentration. However, the relative signal intensity of a given peptide correlates well with the absolute concentration, which is used for relative quantification in proteomics. Based on the m/z's and signal intensities, the plot of which is termed a mass spectrum, the ions can be separated. Due to the naturally occurring C13 isotopes, the mass spectrum will contain m/z signals from a given peptide with only C12, with one C13, two C13, and so forth, thereby generating an isotopic distribution on the mass spectrum with a mass difference of one neutron (1.0087 Da). Based on the measured m/z difference in the isotopic distribution, the charge and mass of the peptide can be calculated (61, 86).

Peptide signals selected for identification are isolated by the mass spectrometer, and fragmented by applying energy, typically in a collision-induced dissociation (CID) process. The mechanisms of CID reactions are incompletely understood, but preferentially the C-N peptide bonds are broken. The m/z signals from the resulting peptide fragments are dependent on the peptide amino acid sequence and modifications, and the signals are measured by the mass spectrometer (61, 86). From the measured peptide fragments, the peptide sequences are determined from which the proteins in the sample can be inferred (87).

Several acquisition methods exist for the selection of peptides for fragmentation and identification. The two widely used methods data dependent acquisition (DDA) and data independent acquisition (DIA) are discussed in the following sections.

#### 3.2.1 Data dependent acquisition

**Figure 4:**
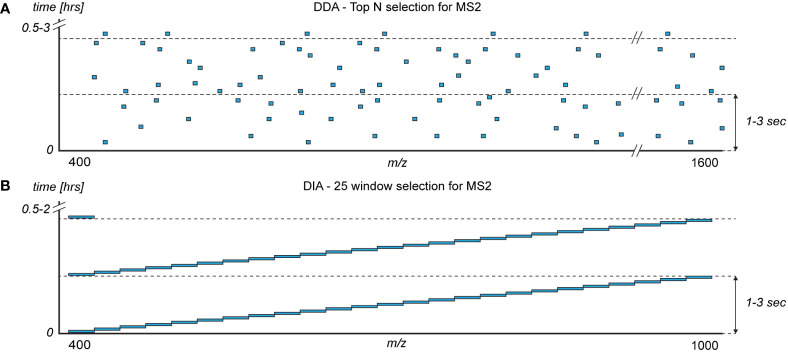
The monitored mass-to-charge (m/z) range (x-axis) of the intact peptides (MS1) during a sample analysis (y-axis) with the two commonly used mass spectrometer acquisition methods for selecting peptides for fragmentation (MS2): **(A)** data dependent acquisition (DDA) and **(B)** data independent acquisition (DIA). Blue boxes represent the typical distribution of m/z windows for selecting peptides for fragmentation and identification.

DDA is arguably the most applied acquisition method in proteomics. However, it is expected that DIA will supersede DDA in the near future. In DDA, the m/z signals of peptides eluting from the column are determined in a mass spectrum termed a MS1. Typically, an m/z range of 400-1600 is monitored, yielding coverage of most peptides generated by trypsin from human samples. Based on the MS1, top N peptides (typically 5-20) are selected for identification based on intensity (**Figure 4A**). One at a time, the selected peptides are isolated, fragmented, and the fragment masses are determined in a mass spectrum termed a MS2. The top N MS2-spectra are proceeded by another MS1 within 1-3 seconds, and the cycle is repeated throughout the analysis which can last tens of minutes to a few hours per sample (88).

The data produced by modern mass spectrometers is a collection of MS-spectra which can be tens of spectra per second of analysis time. The identification of peptides and proteins from database searching was in 1994 automated with SEQUEST, in a landmark publication by Eng, McCormack, and Yates (89). Since then, a number of search engines have been developed, including Mascot, and Andromeda (90) integrated in the widely used MaxQuant software (91). The MS2 m/z signals are matched (**Figure 3C**) to predicted values based on a user-provided protein database which allows for identifying the peptide and protein sequences. Resultingly, proteins which are not present in the database will not be identified even if they are present in the sample. Entire proteomes are often included in the search and uniprot.org is a widely used resource. The protein databases have been established in sequencing studies which have enabled the field of proteomics (63).

Although widely used, DDA is less efficient when analyzing samples with few high abundant proteins such as plasma. During elution of high abundant peptides, the MS1 mass spectrum, which detects the resulting peptides eluting from the LC column in the entire monitored m/z range, will be dominated by individual peptide signals. This reduces the relative signal intensity of all other co-eluting peptides. High abundant peptides may thereby prevent the selection of low-abundant peptides for fragmentation and sequencing in MS2, effectively hindering the identification of lower abundant proteins in plasma. Consequently, during elution of high-abundant peptides the absolute sensitivity of the mass spectrometer is reduced yielding a lower number of identifiable proteins (59).

#### 3.2.2 Data independent acquisition

Several alternatives to DDA have been developed of which DIA (also known as SWATH-MS) (92) arguably is the most wildly used alternative. DIA is based on dividing the monitored m/z range into a number of fixed windows (**Figure 4B**), typically with a width of m/z 20-40. A number of different DIA window acquisition schemes exists which are compared elsewhere (93). Commonly, the monitored m/z-range is reduced in DIA compared to DDA to ensure a fast and sensitive method. However, the impact on the detectable proteins is minimal as e.g. m/z 375−1200 covers 95% of the peptides generated by trypsin from the top 1,000 plasma proteins (4). The mass spectrometer slides through the windows sequentially in a fixed manner, isolating and co-fragmenting all peptides in a given m/z window (**Figure 4B**). Resultingly, during elution of high-abundant peptides only the m/z windows containing the high-abundant eluting peptides have reduced sensitivity, which increases the number of identifiable proteins in plasma (8). Added, the non-deterministic nature of DIA decreases the technical variance in protein identification and quantification (4, 94).

The MS2 mass spectrum obtained with DIA contain multiplexed fragment signals from several peptides, in contrast to MS2 mass spectrum obtained with DDA which mostly contain signals from individual peptides. The multiplexed DIA-data complicates the downstream peptide identification and quantitation (92, 95), and has been a key limitation of DIA until recently. However, over the past decade a number of strategies and software solutions have become available for inferring identifications and relative quantifications from DIA data enabling the methodology (93, 96). Examples include DIA-NN, that exploits deep neural networks for the processing of DIA data (97) and Spectronaut by Biognosys. Recent support of DIA in the widely used LC-MS/MS data analysis software MaxQuant with the incorporation of MaxDIA (98), underlines the development and increasing popularity of DIA. From DIA MS2 mass spectrum, peptide sequences are determined by comparison to previously measured MS2 mass spectrum of individual peptides, collected in project-specific spectral libraries. The spectral libraries are typically established using DDA of pooled samples, and the library size can be expanded by analyzing depleted and/or fractionated plasma, which is discussed in section 3.4. As demonstrated by Bennike et al. (4), expanded spectral libraries allows for analyzing plasma samples using DIA, while making use of depleted and/or fractionated samples in the spectral library. The methodology allows for doubling the number of identifiable plasma proteins and increase the quantitative accuracy as compared to DDA, thereby monitoring a larger fraction of the humoral innate immune system components. Expanded spectral libraries can in this way be used to increase the number of identifiable proteins in the DIA analysis up to a certain point, where reduced benefits have been reported of very large spectral libraries (93).

The recent technical development now allows for inferring peptide identifications directly from DIA data based in spectral library-free analysis, thereby only relying on a user-provided protein database. Library-free analysis is supported by both DIA-NN, Spectronaut, and MaxDIA (93, 97–99). Eventually the need for DDA-generated spectral libraries may be removed altogether. However, library-free analysis requires additional computational time and resources which may pose a limitation for large studies. In addition, the ability of spectral library-free methods to identify novel features remains to be investigated, which speculatively could pose a problem for identification of e.g. novel PTMs.

#### 3.2.3 Identification and filtration

To ensure reliable identifications across various platforms, sample types, and protein databases, identified peptides are filtered using a target-decoy competition approach (**Figure 3C**) (100). In short, the protein database is scrambled by reversing the amino acid sequences and used as a decoy. The measured MS2-spectra are matched against both the target and decoy database. All peptide matches are given a score based amongst others on the number of m/z signals and accuracy, with higher scores representing more confident matches. Matches in the decoy database are expected to occur at random and not be present in the sample, and on average they will have lower scores. A minimum score is then determined where commonly 1% of the list of identifications are decoys, termed the global false discovery rate (FDR). All matches with the minimum score or higher in the target protein database are accepted as being true matches, yielding a list of identifications where a maximum of 1% is expected to be false identifications. The method is largely independent of the score used by the search engine or the proteomics workflow and sample (101, 102). However, although widely used as the default method, the target-decoy approach only controls the average proportion of false discoveries (100), and as shown, additional filtering of proteins may be needed (65).

#### 3.2.4 Quantitation and quality control

Following identification, peptides and proteins can be relatively quantified based on the correlation between the peptide signal intensity and concentration in the sample (**Figure 3D**). The quantification strategies in proteomics can roughly be divided into label-free approaches (including spectral counting, MS1-based, and MS2-based) and stable isotope labeling approaches (including SILAC, iTRAQ, and TMT). Briefly explained, in label-free approaches each sample is analyzed separately by LC-MS/MS and the relative peptide quantities are determined by comparing the signal intensities across the different samples (or with spectral counting the number of MS2 spectra). In labelling approaches, each sample is given a unique chemical or isotopic tag prior to LC-MS/MS analysis. The samples are then mixed and analyzed by LC-MS/MS which, amongst others, reduces the number of samples to be analyzed. The relative peptide quantities in the samples are determined based on the signal intensity of the unique mass-different tags. In both approaches, the peptide quantities are subsequently used to calculate the parent protein abundances. A discussion of quantitation strategies is beyond the scope of this review, but have been extensively compared and discussed elsewhere (103, 104).

Following quantitation, a crucial step is quality control of the data. This is commonly done by visually inspecting the LC-MS/MS raw-data and investigating quality control samples which should be run periodically to ensure system performance. High intensity signals from contaminants, low peptide intensities, and low number of identifications can indicate issues with e.g. sample processing or the LC-MS/MS analysis. In addition, unsupervised statistical methods such as principal components analysis (PCA) are often applied to identify outlying samples and identify batch effects, e.g. by investigating if the data is dependent on the sample processing order. Outlying samples should be critically evaluated and potentially removed following proper argumentation. If batch effects are present, attempts can computationally be made to minimize the impact, e.g. using the R package ComBat (105), given that the experimental design has been made with an appropriate randomization.

Quantitation and quality control may be proceeded by identification of features with a statistically significant difference between two or more groups (e.g. sick *versus* healthy) (**Figure 3D**). Depending on the number of quantified proteins or peptides, the number of performed statistical tests can be vast. Correction for multiple hypothesis testing, by adjusting p-values using e.g. Benjamini-Hochberg (106) or permutation-based FDR control (107), is often paramount to control for false positives (type I errors).

### 3.3 Statistical workflows to identify differentiating proteins

A common and critical step in many proteomics studies is the identification of peptides and proteins with a statistically significant difference between the groups. The t-tests remain widely used in proteomics, presumably due to the robustness, simplicity, and current integration into recognized and widely used data analysis tools, including Perseus (108). However, while highly proven, t-test based approaches do not allow for taking confounding factors such as sex and replicates into account, which often necessitates the merging or subdividing of the dataset, which may have an adverse outcome on the conclusions. Several alternative statistical methods have been proposed to the t-test (109, 110) including linear models (LM) and the extended linear mixed effect models. LM allows for including confounding factors, such as age, sex, and batches. The compatibility with more complex experimental designs can remove the need to subdivide and merge the dataset, thereby allowing for analyzing data from all samples in one analysis. Correlations between samples due to confounding factors or repeated measures can be modelled, which may remove the need to merge e.g. technical replicates prior to analysis. LM-approaches thereby enable the evaluation of flexible hypotheses and interactions beyond a direct comparison between groups (111). LM has been demonstrated to have a higher statistical power to identify proteins with statistically significantly changed levels. This is especially the case with smaller sample sizes (n = 5-15 samples), whereas the statistical power often is more similar to the t-test with higher number of samples (n = 50-100) (109, 110). Resultingly, proteomics studies may often benefit from alternative analytical strategies to the t-test (109–113). The increased biological insights which may be obtained have been demonstrated by the reanalysis of proteomics data with LM approaches, which originally was performed using t-tests. Kirov et al. (114) reanalyzed proteomics data from a study of colon tissue samples from UC patients and controls. Using LM rather than t-tests, additional proteins could be associated with UC. The analysis made it possible to associate degradation of the extracellular matrix in the colon mucosa with UC, and identify neutrophils and NETs as the likely drivers of the degradation. The increased statistical power in the reanalysis to identify additional differentiating proteins compared to the t-test-based analysis likely arose from the technical replicates which did not need merging in the LM-based analysis.

Given the robustness and current implementation into existing tools, the t-test remains easy to apply, whereas the application of LM-approaches to proteomics data mostly remains confined to the R framework. Several packages have been developed in R for analyzing proteomics data using additional statistical methods, including MSstats, Proteus, limma, and lme4, which are compared elsewhere (115). However, most R-packages require some scripting experience. Although the statistical analysis of proteomics data increasingly is moving to R, the lack of implementation in the analytical tools commonly used by the proteomics research community, including Perseus (108), likely represents an obstacle for wider adaptation and may explain the retained popularity of t-test based approaches. Incorporation of alternatives to the t-test into "click-based" proteomics data analysis software which is commonly used by the proteomics society, including Perseus, may help maximize the biological insights which can be obtained from proteomics studies.

### 3.4 Workflows to study the plasma proteome

Proteomics have been applied to measure the levels of humoral innate immune system components in plasma, to identify ongoing immune processes in health and disease. Identification of innate immune system markers in plasma holds great potential to noninvasively guide diagnosis and treatment decisions in inflammatory diseases and cancers. However, as outlined plasma proteomics remains technically challenging due to the proteome composition (**Figure 2**). Various workflows for plasma proteomics have been developed, which will be discussed in this section. In addition, a number of practical considerations have been published by Geyer et al to avoid systematic biases in plasma proteomics studies (116).

#### 3.4.1 Depletion of high abundant proteins

Depletion of high abundant proteins can reduce the dynamic range of concentrations and increase the coverage of the plasma proteome. The targeted depletion of high abundant plasma proteins with antibodies was introduced in 2003 (117), and often depletion of the top 7, 14, or 20 most abundant plasma proteins are applied (56). However, as demonstrated antibody-based depletion schemes typically result in a modest increase in number of protein identification by 25-50% while increasing the required hands-on time during sample preparation (4, 118–120), and care should be taken when interpreting the levels of the proteins targeted for depletion. Non-targeted proteins may also unintentionally bind to the depletion material and be partially depleted (120). In addition, the physiological function of albumin as a protein carrier causes albumin depletion to result in unintentional co-depletion of many other non-targeted proteins, including clinically useful biomarkers (121). However, other studies report of relatively few non-targeted proteins to be depleted using top 7 and 14 approaches (118). In sum, the impact of unintended protein depletion when depleting high abundant plasma proteins remains debated, and depletion strategies remain commonly applied in plasma proteomics to increase the proteome coverage. Validation of findings in non-depleted samples e.g. using antibody-based approaches is recommended, and care should especially be taken when interpreting the levels of the proteins targeted for depletion.

#### 3.4.2 Preanalytical fractionation

Preanalytical fractionation also allows for increasing the proteome coverage of plasma samples by expanding the LC-MS/MS analysis time (4, 58). The sample is fractionated, and each fraction is subsequently analyzed by LC-MS/MS, from which the term prefractionation arises. Prefractionation can be performed before digestion on the protein level or after on the peptide level (58). An example of protein-level prefractionation is the polyacrylamide gels from in-gel digestion, and on the peptide level high pH C18 and strong cation exchange (STX) (122). Using extensive prefractionation techniques, Garay-Baquero et al. (55) identified more than 5000 plasma proteins spanning 11 orders of magnitude in concentration and Keshishian et al. (56) identified 5300 plasma proteins using prefractionation in combination with depletion. The studies demonstrate the impressive complexity of plasma and underline the potential for plasma proteomics in characterizing health and disease. Several plasma workflows with prefractionation have therefore been developed (58). However, prefractionation techniques increase the hands-on time for sample preparation and LC-MS/MS analysis by several factors (4), which may be infeasible in large-scale proteomics studies (4, 56). Accordingly, the study by Garay-Baquero et al. only included 21 samples and Keshishian et al. four samples.

#### 3.4.3 Workflows for large cohorts

Focusing on workflows which are compatible with larger cohorts, in 2016 Geyer et al. (7) reported in a landmark publication on the analysis of nearly 1,300 non-depleted non-prefractionated plasma samples using DDA. By incorporating proteomics data in a spectral library-like manner, 437 proteins could on average be identified per individual. The large number of samples enabled a mapping of the effects of losing weight on the plasma proteome, and included reduced levels of CRP and SAA1, and a reduction in other markers of systemic low-grade inflammation in response to weight loss.

In 2018, Bennike et al. (4) developed a 96-well plate workflow based on MStern digest and DIA with an extended spectral library, which from 1 µL non-depleted plasma allows for monitoring on average 439 plasma proteins per sample. A proof of concept was generated on samples from 62 individuals demonstrated an improved proteome coverage and quantitative accuracy of DIA compared to DDA, and the methodology covered all main complement system components. The methodology was used to assess the impact of total pancreatectomy with islet autotransplantation (TPIAT), a specialized surgical procedure performed to relieve the pain of severe chronic pancreatitis. In TPIAT, the pancreas is removed and the insulin-producing islet cells are isolated, and transplanted into the liver to retain endocrine production. The study of 96 samples collected before and a year after surgery demonstrated that TPIAT reduced the levels of many apolipoproteins and IgM. In 2019 Bruderer et al. (6) used a similar workflow and analyzed 1508 non-depleted plasma samples using DIA, and identified 408 proteins per participant. The dataset enabled characterization of the effects of weight loss and weight maintenance on the proteome, revealing a reduction in low-grade inflammation.

In 2023, Viode et al. (78) developed the PerCA workflow, for acid-based precipitation and depletion of high abundant plasma proteins. The perCA workflow enabled, for the first time, the preparation and analysis of more than 3,000 depleted plasma samples identifying 576 plasma proteins on average per individual using DDA, and 1,343 protein using DIA. The increase in identifications with DIA vs DDA is in accordance with the reported increase in identifications reported by Bennike et al. (4). The vast number of samples enabled detection of severe acute respiratory syndrome coronavirus 2 (SARS-CoV-2)–derived proteins in COVID-19 patient plasma, and the study correlated the SARS-CoV-2 protein abundance with the respiratory status of the patients.

The recent development in plasma proteomics workflows now allows for monitoring more than 1,300 plasma proteins in cohorts with thousands of samples. The increased number of monitorable plasma proteins allows for studying an even greater part of the innate immune system's humoral components. This is of high relevance in studies of health and disease, e.g. the immune system development and inflammatory diseases (123). In cancer, the tumor microenvironment stimulates secretion of pro-inflammatory cytokines, which may inhibit or promote cancer proliferation and metastasis though inflammatory processes, e.g. by secretion of the neutrophil chemoattractant IL-8 that also stimulates NETosis (124). The cytokines are detectable in circulation and thereby holds great potential as noninvasive diagnostics and prognostic cancer biomarkers, which has yet to be fully explored (57). The usage of DIA in combination with acid-based precipitation and depletion of high abundant plasma proteins has set a new standard for the feasible number of analyzable samples. Plasma samples should ideally be analyzed using DIA-based approaches, in combination with LM-approaches for identifying statistically significantly regulated proteins. Synovial fluid is a plasma filtrate and by combining proteomics analysis of synovial fluid with transcriptomics of the synovial membrane, Bennike et al. (65) have demonstrated that the majority of the synovial fluid proteins are plasma derived, with a small fraction secreted directly to the fluid. Given the similar proteome composition of high-abundant proteins in plasma and plasma-derived synovial fluid as demonstrated by Bennike et al. (65), studies of synovial fluid and other plasma-derived body-fluids may likely also benefit from proteomics workflows optimized for plasma. While current workflows providing coverage of the majority of the innate immune system humoral components in workflows compatible with thousands of samples, the concentration of low abundant signaling molecules including cytokines remains below the limit of detection (4, 38, 65, 76). Proteomics workflows enabling determining the levels of cytokines, many of which are APP inducing, would likely cause a paradigm shift, and enable characterization of disease specific cellular signaling and innate immune reactions in inflammatory diseases and cancers.

## 4 Ontogeny of the complement system components in the first week after birth

The innate immune system plays a significant role in providing immunity for newborns (125–127). Plasma proteomics workflows have recently been applied to study the early developmental trajectory of the innate immune system humoral components in the critical first weeks after birth (53, 82). In the mature immune system, a key aspect of immunity to bacterial infections are circulating antibodies against polysaccharides on pathogenic bacterial surfaces. However, coming from a tightly regulated *in utero* environment with tolerance-inducing immune suppression, the newborn immune system is in a uniquely developing state and is naïve to most pathogens. Lacking an efficient adaptive immune response, in the first six months after birth newborns largely rely on the innate immune system for immunity. The immune protection is aided by maternal IgG that is transferred from the mother across the placenta and circulate in the fetus and newborn plasma in the first months after birth until degraded (125–127). In addition, maternal secretory IgA (sIgA) represents over 90% of the Ig-content in human milk and early colostrum, and postnatally sIgA adds to protection against infections, primarily in the intestinal epithelium and do not enter circulation in substantial amounts (128–130).

As outlined in the introduction, the complement system is a vital part of the innate immune system response, working in combination with immune cells. The importance of the complement system for newborn immunity to pathogens is increasingly studied (53, 82, 131). However, few plasma proteomics studies have been performed of the innate immune system development in the critical first week after birth, likely due to the difficulty in obtaining relevant samples and the analytical challenges in analyzing small sample volumes (125, 132, 133). Given the central role of the innate immune system for newborn immunity, this section discusses recent proteomics findings in the development of innate immune system humoral components circulating in plasma in the first week after birth.

### 4.1 Complement system components levels at birth

To enable a functional interpretation of the relative changes to complement components (**Figure 1**) which are provided by plasma proteomics, complement levels in newborns relative to adults will first be described.

The complement components are synthesized early in fetal life, mainly by hepatocytes with a few exceptions including C1q and C7 being secreted mainly from neutrophils and monocytes/macrophages (134). The concentration of most complement components are reduced, with term newborns ranging from 10% to 80% relative to adults (131, 135, 136). A notable exception is C7 from the MAC which is near adult levels (131, 136). Newborn levels of MBL from the lectin activation pathway (**Figure 1B**) and C3 are disputed, with reported levels ranging from near adult levels to approximately 50% (131, 135, 137). The overall deficiency in complement level are even more pronounced in premature newborns (131, 136, 138), and in premature newborns with patent ductus arteriosus, reduced levels of C8 have been shown (139). Resultingly, newborns and in particular premature newborns have a decreased efficiency in bacterial lysis and opsonization, leading to reduced immunity and increased susceptibility to infections (131).

### 4.2 Complement system component levels the first week after birth

As outlined, proteomics can characterize the main complement system components in a plasma sample, and a proteomic study by Bjelosevic et al. (140) has shown that the plasma profiles of neonates are strikingly dissimilar to that of children and adults.

Focusing on the critical first week after birth, a study by Lee et al. (82) analyzed blood from newborns collected on the day of birth and a sample on day 1, day 3 and day 7. The samples were analyzed by proteomics, transcriptomics, metabolomics, and single-cell immunophenotyping. Using "multi-omics" integration the study found that the developmental profiles overall follow a robust trajectory in the first week after birth, even in populations from different geographical areas. The findings underline that after birth the innate immune system undergoes vast, but robust, developmental changes within the first week after birth.

Focusing on the complement system, a re-analysis of the proteomics data by Bennike et al. (53) using LM-approaches enabled a characterization of the developmental trajectory of all main complement components. The study confirmed that levels of complement components mainly related to the classical activation and terminal pathways increase over the first week after birth. Namely, plasma levels of the PRR in the classical activation pathway (**Figure 1A**) C1q, and the other components in the C1-complex (C1r, and C1s) increase significantly as early as day 3. In contrast, plasma levels of the PRR in the lectin activation pathway (**Figure 1B**), FCN-2 and FCN-3 decreased over the first week after birth. Lacking a distinct PRR, the alternative activation pathway (**Figure 1C**) is more difficult to characterize, but levels of CFB increased as early as day 1. In contrast, levels of the central alternative complement enhancer CFP was decreased at day 7. Given the relative deficiency in most complement components, the findings could indicate that the development of the classical activation pathway is the first to be initiated postnatally (53). Supporting these findings, a proteomics study by Grumach et al. (138) found no change to MBL nor CFP levels at day 5.

Focusing downstream of the complement PRRs, the study by Bennike et al. (53) demonstrated that plasma levels of C2 and C4 in the classical and lectin activation pathways increased in the first week after birth and as early as 24 hours after birth. In addition, plasma levels of main complement components in the MAC (C5, C6, C8, and C9) increased whereas plasma levels of C7 decreased. C7 is often the rate-limiting factor in MAC formation and is at relatively high levels at birth (131, 140). The observed increase may indicate the development of components in the classical activation pathway, the terminal pathway, and MAC towards functional levels in the first week after birth.

### 4.3 The classical complement system pathway early after birth

The apparent increase in main components in the classical activation pathway and the terminal pathway in the first week after birth goes against the previous notion that the entire complement system develops as a single unit (141). However, the findings are supported by an increased functional activity of the classical complement pathway in plasma from 5 days old newborns compared to at birth in cord blood (138). The difference in activity was even more pronounced for preterm neonates (< 34 week) where no significant functional activity of the classical pathway could be measured in cord blood but functional activity could be measured at day 5 (138), thereby aligning with the trajectory of the complement component plasma levels.

Speculatively, these findings could indicate a key role for the classical complement pathway in neonatal immunity. The main target molecules of the classical complement pathway PRR are IgG and IgM. IgG is the most abundant Ig in circulation in newborns, infants, and adults (142). Newborn IgG levels are above adult levels, due to active maternal transfer across the placenta medicated by the neonatal Fc receptor (143). IgG exists in subclasses IgG1-4, where IgG1, and IgG3 have the highest classical complement activation potential (22, 144). Nearly mirroring the activation potential, the placental transfer efficiency has been reported to be IgG1>IgG3>IgG4=IgG2 (145) making IgG1 the main maternally transferred Ig-subclass over the placenta and the most abundant in fetal and maternal circulation (142). Bennike et al. (53) have integrated proteomics and transcriptomics data and demonstrated an increased endogenous IgG1 synthesis in newborn whole blood as early as three days after birth. However, the synthesis rates remained insufficient to maintain the high IgG1 plasma levels at birth, caused by the efficient placental transfer of maternal IgG1. Accordingly, IgG levels reportedly decline over the first week after birth (53) and until five months of age (146). In contrast, IgM cannot transverse the placental membrane and newborn levels are low relative to adults (146). Bennike et al. reported that increased plasma IgM levels and endogenous synthesis can be detected in the whole blood cells within the first week after birth (53).

The studies demonstrate the increased plasma concentration of nearly all main components of the classical- and terminal complement pathways early from birth. Speculatively, increasing the classical- and terminal complement pathways to functional plasma concentrations early from birth, would enable newborns to initiate a humoral innate immune response against pathogens that are recognized, for instance, by maternal IgG1. This would highlight the importance of the classical complement pathway for newborn immunity, which evolutionarily is the most recently developed complement pathway compared to the lectin- and alternative pathway (147). Arguing against this hypothesis, higher plasma levels of activation product specific to the alternative pathway has been reported in 6 hour old newborns with early onset infection, compared to the activation product from the classical pathway (148). This could indicate increased activity of the alternative pathway in neonates. However, impaired regulation by the low newborn levels of CFH and CFI, which only increase significantly after the first week after birth (53, 138), may also explain the findings. Additional analysis of the complement levels at a higher time resolution and plasma proteome coverage, using recently developed plasma proteomics workflows, may enable additional insights into the development of the newborn innate immune system in the critical first week after birth.

## 5 Neutrophil extracellular traps in inflammatory diseases

The formation of NETs by neutrophil NETosis represents an important innate immune response to pathogens. In addition, NETs have been correlated to cancer progression and metastasis, and IL-8 secreted by the tumor microenvironment has been found to recruit neutrophils and induce NETosis (124). The role of neutrophils in cancers both as pro- and anti-tumor activity inducing entities is becoming increasingly apparent (14, 123). NET enzymes may degrade the extracellular matrix, in some circumstances inhibiting tumor growth and in others supporting metastasis (14), and NETs have been reported to catch circulating cancer cells and thereby promote metastasis (149). NETs may thereby exhibit both a pro- and anti-tumor activity (14). Proteomics has been applied to study the protein coating on NETs. A study by Chapman et al. has identified 197 proteins on NETs and found that the protein coating is vastly depending on the type of stimulus (150), supplementing the list of 29 NET-associated proteins previously reported by O'Donoghue et al. (43). Citrullinated histone H3 bound to cell-free DNA is today considered a hallmark of most NETs, in addition to neutrophil elastase and MPO (151, 152). However, no NET-unique protein markers nor PTMs have been identified, and proteomics identification is often supported by immunostaining and cell-free DNA measurements. While being an important innate immune reaction providing immunity, NET-formation has also been associated with the pathogenesis of several immune-related diseases, including the prevalent RA (37) and IBD (3). The recent findings will be discussed in this section.

### 5.1 Rheumatoid arthritis and neutrophil extracellular traps

RA is the most common systemic inflammatory joint disease, affecting 0.5%–1% of the population worldwide. The disease is characterized by autoantibodies and immune complexes, leading to neutrophil infiltration of the synovial fluid in the joints and can ultimately result in cartilage damage and joint destruction. In RA, the first described autoantibody was rheumatoid factor (RF) which has affinity to the Fc part of IgG (153, 154). PAD4 citrullination has been associated with RA (151) and anti-citrullinated protein antibodies (ACPA) have been identified in 50–70% of RA patients at diagnosis, with a specificity over 90%. The ACPA have affinity for amongst others citrullinated fibrinogen-alpha, vimentin, and actin (155, 156), and ACPA targeting of citrullinated proteins is thought to drive the inflammation in ACPA positive RA (157).

As shown by several proteomics studies, ACPA targets are present in the synovial fluid from RA patients (37, 38, 158). However, the initial tolerance breach and production of ACPA targeting autoantibodies in RA can be detected years before disease onset, and may take place outside the joints (153). In addition to the synovial joints, several organs have been suggested as the tissue where the initial triggering of tolerance breach in RA occurs, based on the proteomic identification of ACPA targets. Using proteomics, Ytterberg et al. (159) identified joint ACPA targets in the synovial and lung tissue, suggesting the lungs as a site of tolerance breach. Accordingly, the environmental risk factor with the highest impact for RA is smoking, and smoking has been reported to increase PAD2 expression in the lung tissue and citrullination in bronchoalveolar lavage cells (160). However, also mucosal inflammatory processes have been associated with local ACPA production in serum positive individuals without clinical RA (161), and using proteomics Bennike et al. (162) have identified ACPA targets in the colon of RA patients and controls. Given the findings and the heterogenicity and systemic nature of RA, a single pathogenic origin is likely too simplistic to cover the complexity of ACPA positive RA.

Eventually, the disease progresses, and immune cells are stimulated to infiltrate the synovial membrane and synovial fluid, where several studies have associated NETs with RA. Proteomics studies by Birkelund et al. (158) and Spengler et al. (37) have demonstrated that NETs can be detected in the synovial fluid during RA. A proteomics study by Chapman et al. (150) has demonstrated that NETs generated by neutrophils from healthy controls and from RA patients are coated with ACPA targets, including citrullinated histone H3. Supporting this, a proteomics study by Spengler et al. (37) demonstrated that neutrophils isolated from RA synovial fluid express high levels of PADs compared to osteoarthritis (OA) patients, and that NETs in RA synovial fluid are coated with PAD2 and PAD4 enzymes and citrullinated proteins. In addition, the level of cell-free DNA was increased in RA consistent with increased levels of NETs in the synovial fluid. In agreement, the proteomics study by Birkelund et al. (158) found increased levels of NET associated proteins in synovial fluid RA compared to spondylarthritis (SpA) patients, and a correlation was found between cell-free DNA levels and NET proteins.

Speculatively, the increased levels of NETs in RA synovial fluid could be more than the passive result of neutrophil infiltration and activation, but rather have a central part in RA by actively driving the disease through the generation and presenting of ACPAs targets. The increased levels of NETs, and NET-bound ACPA targets within the joint would maintain the ACPA induced inflammation in RA. In addition, PADs bound to NETs could citrullinate proximate proteins which would represent additional ACPA targets within the joint, thereby further maintaining inflammation (37). Supporting this, autoantibodies in the serum of RA patients has been found to react with NETs (163), which highlight the involvement of NETs in the etiology of RA. Accordingly, the therapeutic inhibition of NETosis and increase clearance of NETs are being investigated in inflammatory diseases and cancer, and is discussed in section 5.3.

### 5.2 Inflammatory bowel disease and neutrophil extracellular traps

NETs have also been associated with the disease etiology of IBD (3), a disorder which affects more than 7 million people worldwide with an increasing incidence (164). IBD comprise several chronic disorders of the gastrointestinal tract, where Crohn's disease (CD) and ulcerative colitis (UC) are the most prevalent. IBD result in a reduced quality of life and life expectancy for the patients, and a significant economic burden for society due to lost labor and direct health care expenses (165, 166).

A hallmark of IBD is neutrophil infiltration and inflammation of the intestinal mucosa. The phenotype varies considerably between patients and the etiology and pathogenesis of IBD remains incompletely explained (166). However, there seems to be agreement on the following hypothesis of the IBD etiology; "an aberrant immune response against the gut microbiota, triggered by environmental factors in a genetically susceptible host" (167).

An increasing number of proteomics studies have investigated the IBD colon mucosa using LC-MS/MS based techniques and associated NETs with IBD. First reported in 2015, Bennike et al. (3) observed NETs in the UC colon mucosa, detected using proteomics and visualized in the colon tissue with confocal microscopy, thereby associating NETs with IBD. The abundance of the NET protein S100A9 was found to correlate with the colon inflammation (3). A reanalysis of the dataset by Kirov et al. (114) using LM-approaches identified five-times the number of proteins with significantly different levels in the colon mucosa. The increased amounts of data, allowed for identifying degradation of the colon mucosa extracellular matrix as a key element in UC, including lower levels of elastin and collagen. The degradation is likely driven by the increase of several proteases, including neutrophil collagenase and the NET associated neutrophil elastase. Consistent with the protease-driven extracellular matrix degradation, a comparison to published transcriptomics datasets found increased mRNA levels of elastin and collagen. This could be indicative of a transcriptional compensation, which although compensatory is insufficient to maintain the protein levels and thereby prevent degradation of the extracellular matrix. The findings demonstrate that degradation of the extracellular matrix is part of the UC pathology, and may be driven in part by neutrophils and NETs (114).

The findings have been supported by additional immunohistochemical studies. In 2019, the presence of NETs in UC mucosal tissue was confirmed by Dinallo et al. (168) and in 2020 Li et al. (169) identified NETs in CD, in addition to UC tissues, and increased plasma levels of cell-free DNA. The finding of NETs in CD was confirmed in 2022 by Schroder et al. (170). The studies also confirmed the correlation between NET abundance and degree of inflammation, further supporting the involvement of NETs in the IBD etiology. Dinallo et al. (168) demonstrated that circulating neutrophils isolated from UC patients produce NETs in response to TNF-α stimulation, and reduced expression of NET-related proteins and diminished NET formation were seen in patients receiving successful treatment with anti-TNF-α (168). The findings indicate a role for NETs in sustaining mucosal inflammation in UC and that part of the anti-inflammatory effect of successful IBD treatment could be through reducing NETosis.

### 5.3 Neutrophil extracellular traps as a therapeutic target

NETs have been found to correlate with inflammation and suggested to have an active role in driving chronic inflammation (171). Given the association between NETs and inflammatory diseases, NET targeted therapies are under consideration focused on improving NET clearance or inhibiting NET formation.

During infections, NETs have been found to persist for several days (34). Although the aspects of mechanisms that clear NETs remain incompletely described (172), the degradation by deoxyribonuclease (DNase) I (34) and endocytosis by macrophages have been identified (173). In inflammatory diseases it has been suggested that DNase I is inhibited by anti-NET antibodies, which may block access to the NETs, and cause impairment of NET degradation which results in sustained inflammation (174). Speculatively, this could be relevant in a subset of inflammatory diseases where autoantibodies play a central part, including RA. Supporting this, increased NET degradation through administration of DNase I or inhibition of NETosis decreased cytokine levels in a DSS colitis mouse model of IBD (169). A proteomics study by Fisher et al. (175) found high levels of NETs in of sputum from severe SARS-CoV-2 induced COVID-19 patients, which was confirmed by microscopy. Treatment with DNase reduced the NETs levels and inflammation in the airways, and plasma proteomics revealed a lower systemic inflammation. The findings indicate that NETs may contribute to the respiratory failure in severe COVID-19 patients and NETs targeting therapies using DNase may be efficient. However, following removal of chromatin from the NETs with DNase, active neutrophil elastase and histones persist and may cause inflammation until cleared through unknown mechanisms (176). General inhibition of PADs may therefore be a more efficient strategy, and inhibition of NETosis by Cl-amidine have been shown to reduce the severity of collagen-induced arthritis in mouse models (177, 178). Although the studies did not directly investigate the impact on NET formation, the findings suggest that inhibiting NETs by inhibiting PADs may represent a therapeutic target for the treatment of RA and IBD. Given the pro-tumor activity exhibited by NETs in some tumors, the potential of NET targeting are also investigated as means of therapy, where e.g. Cools-Lartigue et al have shown in a mouse model that treatment with DNase may reduce metastasis slightly (149).

Based on the findings, NETs are currently being investigated as a therapeutic target in IBD (179), RA (179, 180), and cancers (57). Inhibition of NET production and proteolytic activity in PAD4 and neutrophil elastase-deficient mouse models have been found to prevent collateral host tissue damage by NETs (176). While efforts are being made to produce an isotype specific and potent PAD4 inhibitor, currently no PAD4 inhibitor is ready for clinical use (151). Additional characterization of NET formation and clearance may help guide studies focused on novel therapeutic approaches to improve NET clearance or inhibiting NET formation for treatment of various inflammatory diseases and cancers. In addition, identification of innate immune system components, including NETs, released to circulation, with predictive value for diagnosis and prognosis may help guide therapy decisions.

## 6 Conclusion and future directions

Recent advances within proteomics have enabled the increasingly comprehensive characterizing of the proteome, while reducing the analysis and sample preparation time, thus facilitating the analysis of larger cohorts. The development is especially apparent within the technically challenging analysis of blood-plasma, where recently developed workflows enable the monitoring of more than a thousand proteins in more than three thousand samples. Additional studies of plasma, and samples with similar challenges such as synovial fluid, may benefit significantly from DIA-based methodologies as the default analytical strategy. The development of plasma proteomics workflows compatible with large cohorts, with sufficient sensitivity to determine the levels of low abundant cytokines, would likely cause a paradigm shift and enable characterization of disease specific cellular signaling and innate immune reactions. Non-invasive identification of biomarkers with diagnostic and prognostic value holds great promise for guiding treatment decisions for inflammatory diseases and cancers. The potential of integrating data from several different omics including proteomics, transcriptomics, and metabolomics in multi-omics approaches to obtain unique biological insights, have been demonstrated in several studies, highlighting their value in understanding the innate immune system in health and disease. The recently developed plasma proteomics workflows hold great potential for characterizing the innate immune system in health and disease. Additional studies of the critical early phase after birth where the innate immune system undergoes vast changes will likely further increase our understanding of the early innate immune system development. Similarly, the association between NETs and several inflammatory diseases and cancer is becoming increasingly evident. A promising avenue under investigation is the development of therapies that either inhibit NET formation or enhance NET clearance. Additional studies into the formation, composition, function, and clearance of NETs, which likely exhibit greater heterogeneity than currently recognized, seem promising in advancing the development of novel NET targeting strategies. These approaches hold potential for mitigating the detrimental effects of NETs and reducing inflammation associated with these diseases. The continued development of proteomics platforms and workflows will continue to offer valuable insights into the proteome and advance our understanding of health and disease with translational perspectives for diagnostics and therapy.

## Author contributions

TB: Conceptualization, Data curation, Visualization, Writing – original draft, Writing – review & editing.
